# The Role of Dietary Antioxidants and Their Potential Mechanisms in Alzheimer’s Disease Treatment

**DOI:** 10.3390/metabo13030438

**Published:** 2023-03-17

**Authors:** Emily Knight, Thangiah Geetha, Tom L. Broderick, Jeganathan Ramesh Babu

**Affiliations:** 1 Department of Nutritional Sciences, Auburn University, Auburn, AL 36849, USA; 2 Boshell Metabolic Diseases and Diabetes Program, Auburn University, Auburn, AL 36849, USA; 3 Laboratory of Diabetes and Exercise Metabolism, Department of Physiology, Midwestern University, Glendale, AZ 85308, USA

**Keywords:** Alzheimer’s disease, antioxidants, coenzyme Q, curcumin, melatonin, probiotics, resveratrol, rosmarinic acid, selenium, vitamin E

## Abstract

Alzheimer’s disease (AD) is a progressive neurodegenerative disorder associated with cognitive decline and characterized by amyloid-β plaques and neurofibrillary tau tangles. Although AD’s exact pathophysiology remains unclear, oxidative stress is known to play a role in the neurodegenerative process. Since no curative treatment exists, antioxidants represent a potential treatment for AD due to their ability to modulate oxidative stress. Therefore, this review aims to examine the impact of antioxidant supplementation and its potential mechanisms on cognitive function. The review primarily discusses research articles published between 2012 and 2022 reporting the results of clinical trials involving antioxidant supplementation on cognitive function in individuals with AD. Antioxidant supplementation included probiotics, selenium, melatonin, resveratrol, rosmarinic acid, carotenoids, curcumin, vitamin E, and coenzyme Q. While the studies included in this review did not provide much evidence for the beneficial role of antioxidant supplements on cognitive function in AD, the results varied from antioxidant to antioxidant and among trials examining the same antioxidant. Furthermore, many of the studies’ findings face several limitations, including short trial durations, small sample sizes, and a lack of diversity among study participants. As a result, more research is required to examine the impact of antioxidant supplementation on cognitive function in AD.

## 1. Introduction

Globally, 697 individuals over the age of fifty suffer from dementia per 100,000 persons, with the prevalence of Alzheimer’s Disease (AD) being 324 per 10,000 [1]. In Europe, an estimated 7.1% of the population has AD, with the prevalence being higher in females than males and in individuals with an older age [2]. In 2020, more than 6 million adults in the United States had AD, with the prevalence forecast to exceed 13 million by 2060 [3]. During 2020, the healthcare costs associated with AD in the United States reached $305 billion, and the economic burden is expected to increase with the aging population [4]. AD affects an individual’s quality of life and that of those who care for them [4,5,6]. As a result, treating AD is critical to improving the health and well-being of individuals with AD and those who care for them.

AD is a multifactorial neurodegenerative disorder associated with a gradual decline in cognitive function and characterized by extracellular amyloid-β (Aβ) plaques and intracellular neurofibrillary tau tangles (NFT) [7,8]. Since years may pass before the onset of neurodegeneration leads to the development of cognitive decline, AD represents a continuum in which an affected individual progresses from normal cognitive function to mild cognitive impairment (MCI) and, finally, dementia [8,9,10]. Since a definitive diagnosis of AD cannot be made without an autopsy, establishing biomarkers for diagnosing AD, particularly in the early phases of the disease, is critical for early diagnosis and detection [7]. For example, several trials indicated that cerebral spinal fluid (CSF) Aβ_42_ levels decreased and tau levels increased with disease progression [11].

While Aβ plaques and NFT represent the histopathological hallmarks of AD, they may be the result of other neurodegenerative processes and not the initiating factor in AD [9]. The formation of Aβ plaques involves the cleavage of the single transmembrane protein, amyloid precursor protein (APP) [12]. In the non-amyloidogenic pathway, APP is cleaved by α-secretase to soluble APPα, while in the amyloidogenic pathway, APP is cleaved by β-secretase and γ-secretase to Aβ [12,13,14]. Mutations in presenilin-1 (PS1) and PS2, the catalytic subunits of γ-secretase, are associated with increased Aβ production [14]. NFTs are primarily composed of hyperphosphorylated tau, a microtubule-associated protein that can interact with other hyperphosphorylated tau proteins to form paired helical filaments [7,15]. Nevertheless, the exact role of Aβ and NFT in the pathophysiology of AD remains unclear.

Although the exact pathophysiology of AD is uncertain, mechanisms involved in neurodegeneration may include oxidative stress [7]. Oxidative stress occurs when the generation of reactive oxygen species (ROS) and reactive nitrogen species exceeds the body’s antioxidant defense systems [16,17,18,19]. Aging is associated with a decline in antioxidant enzymes and increased oxidative stress and inflammation, which may contribute to the development of chronic diseases [20]. Compared to other cells in the body, neurons possess more polyunsaturated fatty acids in their cell membranes, and their higher metabolic rate results in greater oxygen consumption [21]. As a result, the brain is particularly vulnerable to oxidative stress, which can damage lipids, proteins, and deoxyribonucleic acid (DNA) in cells [19,21,22]. Moreover, higher levels of serum malondialdehyde (MDA), a marker of lipid peroxidation, are observed in individuals with AD compared to healthy controls [23]. In addition to lipid, protein, and DNA damage, oxidative stress may contribute to the aggregation and misfolding of Aβ plaques and NFT [24]. There are several potential sources of ROS in AD. Research indicates that Aβ plaques, mitochondrial dysfunction, metal dyshomeostasis (iron, copper, and zinc), and inflammation may contribute to the generation of ROS [21,25,26,27,28,29]. For example, copper and iron can accumulate in Aβ plaques and contribute to the generation of ROS [25]. Additionally, neuroinflammation involves microglia activating nicotinamide adenine dinucleotide phosphate oxidase, which also generates ROS [27]. Overall, oxidative stress contributes to neuron damage and cell death [24]. Therefore, oxidative stress plays an important role in the neurodegeneration associated with AD.

Currently, there is no cure for AD, and treatment focuses on alleviating symptoms. Therapeutic drugs include cholinesterase inhibitors (donepezil, rivastigmine, and galantamine), N-methyl-D-aspartate receptor antagonists (memantine), and anti-Aβ monoclonal antibodies (aducanumab) [30,31,32,33]. During the pathophysiology of AD, cholinergic neuron death is thought to contribute to the decline in cognitive function [34]. Cholinesterase inhibitors function by preventing the enzyme acetylcholinesterase from breaking down the neurotransmitter acetylcholine [31,34,35]. As a result, more acetylcholine remains in the synaptic cleft, thus improving cholinergic neuron activity and cognitive function [34,36]. In AD, N-methyl-D-aspartate receptors are thought to be overactivated by the neurotransmitter glutamate, making it difficult for the brain to distinguish normal glutamate signaling [37]. N-methyl-D-aspartate receptor antagonists may operate by reducing the activation of the receptors by glutamate, thus improving cognitive function [37]. In addition to only alleviating AD symptoms, cholinesterase inhibitors and N-methyl-D-aspartate antagonists are associated with adverse side effects (decreased appetite, diarrhea, dizziness, insomnia, and nausea) and drug interactions [31,38]. Anti-Aβ monoclonal antibodies are thought to facilitate Aβ clearance from the CSF [32,33]. The Food and Drug Administration recently approved the anti-Aβ monoclonal antibody, aducanumab, for use in mild AD treatment [32,33]. Despite data indicating that aducanumab reduces Aβ plaques, current data on the impact of aducanumab on cognitive function is inconclusive, and the drug is associated with cerebral edema and intracerebral hemorrhages [32,33]. Given the limitations of the current treatments for AD, more research is needed to identify and develop strategies for modifying disease progression.

Since oxidative stress is involved in the pathophysiology of AD, antioxidants represent a potential treatment for AD. A previous review by Pritam et al. [39] discussed a wide range of antioxidants with therapeutic potential in AD but lacked information on the results of recent clinical trials. Therefore, this review aims to examine the impact of antioxidant supplementation and its potential mechanisms on cognitive function in individuals with AD. PubMed searches were used to identify articles published in English between 2012 and 2022, reporting the results of clinical trials that involved antioxidant supplementation in individuals with AD and included cognitive function as an outcome. The following review discusses the results of clinical trials involving supplementation with probiotics [40,41,42,43], selenium [44,45,46], melatonin [47], resveratrol [48,49,50,51], rosmarinic acid [52], carotenoids [53,54,55], curcumin [56], vitamin E [57,58,59,60], and coenzyme Q [58] (Table 1).

## 2. Probiotics

According to the Food and Agriculture Organization of the United Nations and the World Health Organization, the term ‘probiotics’ refers to “live microorganisms that, when administered in adequate amounts, confer a health benefit on the host” [61,62]. The beneficial role of probiotics stems from the ability of bacteria to modify the composition and, thus, the activity of the gut microbiome (the microorganisms residing along the host’s gastrointestinal tract) [63,64]. The ability of the gut microbiome to communicate with the central nervous system via the gut-brain axis has led to an interest in using probiotics to treat neurological diseases [65,66,67]. Additionally, a systematic review and meta-analysis by Hung et al. [68] observed a significant reduction in the diversity of the gut microbiome of individuals with AD compared to healthy controls. Furthermore, pathogenic microorganisms may contribute to the pathophysiology of AD through the generation of lipopolysaccharides and bacterial amyloid fiber [69,70]. The beneficial effects of probiotics can depend on their strain, but research has linked some species of probiotic bacteria to antioxidant defense [71]. For example, administering *Lactobacillus reuteri*, *Lactobacillus rhamnosus*, and *Bifidobacterium infantis* alleviated oxidative stress and inflammation in rats treated with Aβ_1–40_ [72]. Therefore, probiotics with potential antioxidant functions are of interest for possibly treating AD. Four studies [40,41,42,43] examined the impact of probiotic supplementation among individuals with AD.

In Iran, Akbari et al. [40] examined the consumption of 200 mL/day of milk either with (*n* = 30) or without (*n* = 30) probiotic supplementation (2 × 10^9^ CFU/g of *Lactobacillus acidophilus*, *Lactobacillus casei*, *Lactobacillus fermentum*, and *Bifidobacterium bifidum*) for 12 weeks in individuals with AD. After treatment, the probiotic group experienced a significant improvement in cognitive function compared to the control group, as indicated by an increase in Mini-Mental State Examination (MMSE) scores [40]. Additionally, the probiotic group experienced a significant improvement in high-sensitivity C-reactive protein (hs-CRP), the homeostatic model of assessment of insulin resistance (HOMA-IR), the homeostatic model of assessment for B-cell function (HOMA-B), MDA, the quantitative insulin sensitivity check index (QUICKI), triglycerides, and very low-density lipoprotein [40]. As a result, the study indicated that probiotic supplementation might improve cognitive function among individuals with AD by improving the lipid profile and decreasing insulin resistance and oxidative stress. However, no significant differences between the two groups were observed in total antioxidant capacity (TAC), glutathione, nitric oxide (NO), fasting blood glucose, low-density lipoprotein, high-density lipoprotein, total cholesterol, or body mass index after 12 weeks of treatment [40]. The study’s small sample size, short time frame, and lack of data on the host’s microbiome before and after supplementation limit the study’s results.

Furthermore, in Iran, Agahi et al. [41] examined the effects of probiotic supplementation on cognitive function using the Test Your Memory (TYM) test modified for an Iranian population. Men and women with AD were randomized to receive either a placebo (*n* = 30) or a probiotic (*n* = 30) for 12 weeks [41]. The probiotic group received two capsules every other day, each containing 3 × 10^9^ CFU of bacteria. The first capsule contained *Lactobacillus fermentum*, *Lactobacillus plantarum*, and *Bifidobacterium lactis*, while the second capsule contained *Lactobacillus acidophilus*, *Bifidobacterium bifidum*, and *Bifidobacterium longum* [41]. At the end of 12 weeks, 23 participants remained in the control group and 25 in the probiotic group and were included in the final analysis [41]. After 12 weeks of treatment, statistical analysis indicated a significant difference between the two groups for markers of oxidative stress (TAC, MDA, and NO), but post hoc analysis made these differences negligible [41]. Additionally, no significant differences were observed between the two groups for cognitive function (TYM), markers of inflammation (tumor necrosis factor-α (TNF-α), interleukin-6 (IL-6), IL-10, or other markers of oxidative stress (total glutathione and 8-hydroxy-2′-deoxyguanosine) [41]. As a result, the study does not support the role of probiotics in improving cognitive function or markers of inflammation and oxidative stress in AD. Nevertheless, the authors [41] noted that the difference in the study results with those of Akbari et al. [40] could be accounted for by the different supplements and the higher percentage of participants with severe AD.

In Austria, Leblhuber et al. [42] examined the impacts of probiotic supplementation on cognitive function and inflammation. Twenty individuals with AD received 3 g of a probiotic supplement (*Lactobacillus casei*, *Lactococcus lactis*, *Lactobacillus acidophilus*, *Bifidobacterium lactis*, *Lactobacillus paracasei*, *Lactobacillus plantarum*, *Bifidobacterium lactis*, *Bifidobacterium bifidum*, and *Lactobacillus salivarius*) once daily for four weeks [42,73]. After the study, paired serum samples were available for 15 participants, and paired stool samples were available for 18 participants [42]. After four weeks of treatment, no significant changes were observed in cognitive function (MMSE, clock drawing test) [42]. Although the lack of change in cognitive function may indicate that the probiotic supplementation delayed cognitive decline, the study lacked a control group, and four weeks may not be sufficient for observing changes in cognitive function. While analysis of fecal ribonucleic acid (RNA) indicated that the concentration of *Faecalibacterium prausnitzii* increased after four weeks of treatment, no changes were observed for *Clostridium* cluster I or *Akkermansia muciniphila* RNA [42]. Additionally, a significant decline in fecal zonulin was observed, indicating a possible improvement in gut permeability [42]. Although no significant changes were observed in the ratio of serum kynurenine/tryptophan (an indicator of tryptophan breakdown), the levels of serum kynurenine increased, indicating a potential stimulation of the body’s immune system [42]. Despite the study being limited by its small sample size, short trial duration, and lack of a control group, Leblhuber et al.’s [42] data indicate that probiotic supplementation may modulate the composition of the gut microbiota and impact systemic inflammation.

In Brazil, Ton et al. [43] examined the impact of 90-day supplementation with kefir-fermented milk containing *Acetobacter aceti*, *Acetobacter* sp., *Candida famata*, *Candida krusei*, *Enterococcus faecium*, *Lactobacillus delbrueckii delbrueckii*, *Lactobacillus fermentum*, *Lactobacillus fructivorans*, *Lactobacillus kefiranofaciens*, and *Leuconostoc* spp. in individuals with AD taking 10 mg of donepezil. While 16 individuals with AD were initially enrolled in the study and received the intervention (2 mL/kg/day), only 13 completed the study and were included in the final analysis [43]. After 90 days of treatment, Ton et al. [43] observed a significant improvement in cognitive status (MMSE). In addition to examining the impact of probiotic consumption on cognitive function, the impact on measures of inflammation and oxidative stress using blood samples was examined [43]. Probiotic supplementation improved inflammation as indicated by a significant decline in TNF-α, IL-8, and IL12p70 (proinflammatory cytokines), but no change in IL-1b and IL-6 (proinflammatory cytokines) or IL-10 (anti-inflammatory cytokine) levels were observed [43]. Furthermore, serum oxidative stress decreased, as indicated by a decline in ROS (superoxide anion, hydrogen peroxide, and peroxynitrite/hydroxyl radical), a decline in advanced oxidative protein products, and an increase in NO [43]. While the study by Ton et al. [43] indicated that probiotic supplementation could potentially improve cognitive function through a reduction in inflammation and oxidative stress, the results of this study are limited by the small sample size and lack of a control group. Additionally, changes in serum inflammation and oxidative stress markers may not represent changes in brain tissue. The study also lacked data on the composition of the host’s microbiome before and after treatment. Nevertheless, the study supports the potential benefits of probiotic consumption in AD.

While both the studies by Agahi et al. [41] and Leblhuber et al. [42] observed no impact of probiotic supplementation on cognitive function, the studies by Akbari et al. [40] and Ton et al. [43] observed that probiotic supplementation could improve MMSE scores and provided some insight into the potential mechanisms behind this improvement. While Akbari et al. [40] observed no change in serum NO levels, Ton et al. [43] observed a significant increase in NO levels. As mentioned above, the beneficial effects of probiotics may be specific to some bacterial species [71]. As a result, the different probiotic compositions could account for the differences between the two studies. While NO contributes to vasodilation, inhibits platelet aggregation, and functions as a neurotransmitter, it also plays a role in inflammation and can damage cells, particularly after reacting with superoxide anions to generate peroxynitrite anions [74]. Therefore, the impact of increased NO levels on AD in the study by Ton et al. [43] is unclear. Both studies observed a potential beneficial mechanism through the reduction of inflammation, with Ton et al. [43] reporting a decline in TNF-α, IL-8, and IL12p70 and Akbari et al. [40] noting a decline in hs-CRP. Additionally, both studies observed a potential beneficial mechanism through the reduction of oxidative stress, with Ton et al. [43] observing a decline in superoxide anion, hydrogen peroxide, and peroxynitrite/hydroxyl radical and Akbari et al. [40] documenting a decline in MDA. These results agree with preclinical research indicating that probiotics may improve cognitive function in rodent models of AD by reducing oxidative stress and inflammation. In rats who received an intrahippocampal injection of Aβ_1–40_ followed by probiotic supplementation with *Lactobacillus reuteri*, *Lactobacillus rhamnosus*, and *Bifidobacterium infantis*, Mehrabadi and Sadr [72] observed a significant improvement in cognitive function along with a decline in MDA, IL-1, and TNF-α. Furthermore, probiotics may improve cognitive function in AD by decreasing insulin resistance. Akbari et al. [40] observed a reduction in insulin resistance, which may have also contributed to improvements in cognitive function. Overall, two of the four studies support the beneficial potential of probiotics in improving cognitive function in AD.

## 3. Selenium

Selenium, an essential trace mineral, can be obtained from several dietary sources, including Brazil nuts, bread, broccoli, cereals, meat, fish, eggs, milk, and dairy [75]. In the form of selenomethionine and the amino acid selenocysteine, selenium is a component of several proteins in the body. These selenium-containing proteins are known as selenoproteins. Selenoproteins play a critical role in cognitive and thyroid function, along with antioxidant defense [75,76]. For example, glutathione peroxidase 4 (GPX4) is essential in regulating ferroptosis and reducing lipid peroxides [76]. A meta-analysis by Reddy et al. [77] indicated that serum selenium levels are significantly lower in individuals with AD, and the difference remained after controlling for age. Among individuals with AD, a direct correlation existed between serum selenium levels and GPX levels [77]. The prevention of AD by vitamin E and selenium trials observed no significant differences in AD risk among males over 60 supplemented with vitamin E (400 IU), selenium (200 μg), or both in combination or alone compared to a placebo for 5.4 years [78]. Conversely, a six-month clinical trial among individuals with MCI indicated that daily supplementation with Brazil nuts containing 288 μg of selenium improved erythrocyte GPX levels and verbal fluency [79]. As a result, selenium is a compound of interest in possibly treating and preventing AD due to its role in antioxidant defense. Two clinical trials were identified examining the impact of selenium supplementation on cognitive function in AD.

The first clinical trial examined the impact of two different doses of selenium. In Australia, researchers conducted a double-blind, placebo-controlled trial examining the impact of sodium selenate (Na_2_SeO_4_) supplementation among individuals with mild-to-moderate AD [44,45]. Participants were randomized to consume either supranutrional (10 mg sodium selenate; *n* = 20), nutritional (0.32 mg sodium selenate; *n* = 10), or a placebo (*n* = 10) three times a day for 24 weeks [44,45]. The study by Maplas et al. [44] focused on reporting the trial’s primary outcomes (safety and tolerability), while Cardoso et al. [45] detailed the results of an exploratory analysis. Four participants withdrew from the study due to a skin rash (supranutritional group), nail changes (supranutritional group), a diagnosis of normal pressure hydrocephalus (control group), and a change in medications (control group); the control group was composed of participants receiving either nutritional sodium selenate or the placebo [44]. Maplas et al. [44] observed drug-related adverse events (decreased appetite, dizziness, fatigue, headache, lethargy, myalgia, muscle spasms, nail disorder, and nausea) among 68% of study participants. Furthermore, Maplas et al. [44] reported only one severe adverse event (pre-syncope) in a member of the supranutritional group. With 16 members of the supranutritional group and 11 members of the control group experiencing drug-related adverse events, the authors concluded that supplementation with supranutritional selenate for 24 weeks was tolerable and safe [44]. Analysis of secondary variables revealed no significant changes in CSF phosphorylated tau, CSF total tau (t-tau), CSF Aβ_1–42_, MMSE, Alzheimer’s Disease Assessment Scale-cognitive (ADAS-Cog), controlled oral word association test (COWAT), category fluency test (CFT), one-card learning memory task (OCL), identification reaction time task (IDN), and detection reaction time task (DET) [44]. As a result, future studies with larger sample sizes and longer durations may reveal that sodium selenate can improve AD biomarkers and cognitive function.

The exploratory analysis by Cardoso et al. [45] expanded upon the trial’s results by examining the impact of sodium selenate supplementation on plasma and CSF selenium. While plasma and CSF selenium levels increased among participants receiving both nutritional and supranutritional doses, the increase in serum selenium only corresponded to a 3% increase in CSF levels [45]. Furthermore, serum and CSF selenium levels were highly variable within each experimental group [45]. As a result, Cardoso et al. [45] examined study data by regrouping participants as ‘responsive’ and ‘non-responsive’ to sodium selenate based on an increase in serum (responsive, *n* = 17; non-responsive, *n* = 18) and CSF (responsive, *n* = 12; non-responsive, *n* = 14) selenium three times that of baseline levels. There were no differences in the number of drug-related adverse events between the responsive and non-responsive groups [45]. Compared to CSF non-responsive individuals, participants in the CSF responsive group did not experience a significant decline in the MMSE. No significant differences emerged for ADAS-Cog, COWAT, CFT, OCL, IDN, and DET [45]. Nevertheless, the lack of significance in measures of cognitive function could still be accounted for by the study’s small sample size and short duration. Overall, Cardoso et al. [45] highlighted the importance of examining changes in serum and CSF levels of selenium in future studies.

The effects of selenium supplementation and other therapeutic compounds have been investigated. In Iran, Tamtaji et al. [46] examined the role of selenium (200 μg/day) with and without a probiotic (2 × 10^9^ CFU/day of *Lactobacillus acidophilus*, *Bifidobacterium bifidum*, and *Bifidobacterium longum*) supplement among institutionalized individuals with AD. Participants were randomized to consume selenium and a probiotic (*n* = 30), selenium-only (*n* = 30), or a placebo (*n* = 30) for 12 weeks [46]. Following participant withdrawal due to personal reasons, the final analysis included 27 participants in the combination group, 26 in the selenium-only group, and 26 in the placebo group [46]. Similar to Malpas et al. [44], Tamtaji et al. [46] observed no significant difference in MMSE scores between the selenium-only and placebo groups. Malpas et al. [44] estimated that a sample size of 352 participants would be necessary to detect changes in MMSE scores using an 80% power level at a *p*-value less than 0.05. Since the trial by Tamtaji et al. [46] lasted for 12 weeks less than that of Malpas et al. [44], future clinical trials with more extended periods and a greater number of participants may reveal different results. Tamtaji et al. [46] also observed a significant improvement in the MMSE among participants supplemented with both selenium and probiotics. As a result, the combined actions of both selenium and probiotics could improve cognitive function among individuals with AD. Considering the trials by Akbari et al. [40] and Ton et al. [43] that observed a significant improvement in MMSE scores following the consumption of probiotics, future clinical trials should consider adding a probiotic-only group to assess whether the improvement in the selenium and probiotics group results solely from the addition of the probiotic. While the impact of selenium supplementation in individuals with AD remains unclear, future research may clarify the role of selenium supplementation in AD.

The mechanism behind selenium’s potential beneficial role in AD is currently unclear. One possible explanation is that a decline in the body’s selenium levels corresponds to a decrease in selenoproteins that protect against oxidative stress in the central nervous system [77]. For example, Sharma et al. [80] observed that the mice receiving a selenium-deficient diet had significantly lower selenium levels in the cortex and hippocampus than the mice receiving a selenium-adequate diet. Selenium deficiency also decreased GPX activity in the mouse cortex, hippocampus, and cerebellum and increased lipid peroxidation in the cortex [80]. Compared to selenium-adequate mice, the selenium-deficient mice displayed anxiety-like behavior (open field test) and decreased spatial memory (Morris water maze) [80]. As a result, increasing selenium levels through supplementation may reduce oxidative stress and improve cognitive function. Tamtaji et al. [46] observed that selenium supplementation increased serum glutathione levels and TAC. Additionally, after grouping participants as selenium-responsive and non-responsive, a significant improvement in MMSE scores emerged among individuals with AD supplemented with selenium [44,45]. As a result, inadequate selenium levels may contribute to cognitive decline in AD.

The mineral’s role in ferroptosis further supports the importance of adequate selenium. Ferroptosis is an iron-dependent form of programmed cell death in which excess lipid peroxidation, particularly of polyunsaturated fatty acids in the lipid bilayer, leads to loss of membrane integrity, organelle rupturing, and cell death [81]. GPX4, using reduced glutathione (GSH) as a cofactor, protects cells against ferroptosis by reducing lipid hydroperoxides to lipid alcohols [82,83]. Iron dyshomeostasis can participate in the Fenton reaction by increasing intracellular free iron, leading to an increase in oxidative stress and lipid peroxidation. Compared to mice receiving a selenium-adequate diet, those receiving a selenium-deficient diet showed increased iron levels and neuronal loss in their cortex and hippocampus [80]. Additionally, magnetic resonance imaging (MRI) indicated elevated iron levels in the cortex of individuals with AD compared to age-matched healthy controls [84]. As a result, inadequate selenium decreases GPX4 and GSH levels, decreasing the cell’s resistance to ferroptosis.

As seen in the study by Tamtaji et al., the beneficial role of selenium supplementation in AD [46] may be partially accounted for by the inclusion of probiotic supplementation. In this study [46], both the combination group and the selenium-only group experienced a significant improvement in markers of inflammation (hs-CRP), oxidative stress (glutathione), and insulin resistance (insulin, HOMA-IR, and QUICKI). Nevertheless, compared to both the placebo and selenium-only groups, the combination group experienced a significant improvement in cognitive function (MMSE), inflammation (hs-CRP), oxidative stress (TAC and glutathione), and insulin resistance (insulin, HOMA-IR, and QUICKI). Previously, the results by Akbari et al. [40] and Ton et al. [43] observed that probiotic supplementation could improve cognitive function (MMSE), inflammation (hs-CRP, TNF-α, IL-8, and IL12p70), oxidative stress (MDA and ROS), and insulin resistance (HOMA-IR and QUICKI). While these two studies support the potential ability of probiotics to contribute to the improvement in cognitive decline seen in the combination group, the type of probiotic bacteria may influence their mechanisms in human health. A study by Kim et al. [85] observed that the consumption of a probiotic containing *Bifidobacterium bifidum* and *Bifidobacterium longum* for 12 weeks compared to a placebo led to an improvement in cognitive function among community-dwelling older adults. Since the exact contribution of probiotics to cognitive function in AD is unclear [46], future studies should add a probiotic-only group to understand the beneficial changes in this study.

## 4. Melatonin

Melatonin (n-acetyl-5-methoxytryptamine) is a peptide hormone secreted by the pineal gland and is involved in regulating the circadian rhythm [86,87,88]. A systematic review by Nous et al. [89] indicated lower nighttime melatonin levels in the plasma and CSF of individuals with AD. Furthermore, the presence of sleep disorders in individuals with AD increases the risk of early institutionalization [90]. As a result, there is increased interest in using melatonin for the possible treatment of AD. Melatonin supplements include prolonged-release melatonin and fast-release melatonin. Compared to fast-release melatonin supplements, prolonged-release melatonin extended the time plasma melatonin levels remained above basal levels from three to four hours to five to seven hours [91,92]. In addition to increasing the risk of early institutionalization, disruptions of circadian rhythms may contribute to the generation of oxidative stress in AD [90]. As a result, melatonin’s antioxidant properties, particularly its free radical scavenging activity, could play a role in treating AD by reducing oxidative stress [88,93]. Rondanelle et al. [94] observed a significant improvement in MMSE scores among individuals with MCI supplemented with 5 mg melatonin in the presence of 720 mg docosahexaenoic acid (DHA), 286 mg eicosapentaenoic acid (EPA), 16 mg vitamin E, 160 mg soy phospholipids, and 95 mg tryptophan for 12 weeks compared to a placebo. Therefore, melatonin is a compound of interest in treating AD due to its antioxidant activity and ability to regulate circadian rhythms. One clinical trial was identified that examined the impact of melatonin supplementation on cognitive function in AD.

Wade et al. [47] examined the effect of prolonged-release melatonin among individuals with mild-to-moderate AD treated with acetylcholinesterase inhibitors. Participants were randomized to consume either a 2 mg melatonin supplement (*n* = 39) or a placebo (*n* = 34) daily for 24 weeks following a 2-week placebo run-in period and to precede a 2-week placebo run-out period [47]. All enrolled participants were included in the safety analysis [47]. No significant differences were reported between the melatonin (*n* = 8) and placebo (*n* = 2) groups for patient-reported drug-related adverse events [47]. Furthermore, none of the severe adverse events (melatonin group, *n* = 2; placebo group, *n* = 5) that occurred during the trial were study-related [47]. As a result, Wade et al. [47] concluded that supplementation with 2 mg of melatonin for 24 weeks in individuals with AD was safe and tolerable. After excluding participants who withdrew from the trial, 31 participants in the melatonin group and 29 in the placebo group were included in the final analysis [47]. Though the placebo group experienced no significant changes in overall sleep scores as measured by the Pittsburgh Sleep Quality Index (PSQI), the melatonin group experienced an improvement in overall sleep scores, as indicated by a significant decrease in PSQI scores [47]. These results agree with Cruz-Aguilar et al. [95,96], who observed that melatonin (5-mg, fast-release) supplementation had a beneficial impact on sleep among individuals with mild-to-moderate AD (*n* = 8) by reducing sleep latency. In contrast, an earlier clinical trial observed no significant differences in sleep measures using wrist actigraphy among individuals with AD supplemented with either 2.5 mg prolonged-release melatonin, 10 mg fast-release melatonin, or a placebo for eight weeks [97]. While neither the placebo nor melatonin group in the study by Wade et al. [47] experienced a significant change in ADAS-Cog scores, the melatonin group experienced significantly less decline in MMSE and an increase in Instrumental Activities of Daily Living (IADL) compared to the placebo group. After 24 weeks, neither the placebo nor the melatonin group experienced a significant change in the Neuropsychiatric Inventory (NPI) [47]. As a result, Wade et al. [47] concluded that prolonged-release melatonin might improve cognitive function in individuals with AD due to improved sleep efficacy. Nevertheless, further studies are needed to examine the relationship between melatonin and AD, including studies with larger sample sizes and longer durations.

While the exact mechanism of melatonin in AD remains unclear, melatonin may play a beneficial role in AD by improving circadian rhythms and reducing inflammation, oxidative stress, and the formation of Aβ plaque and NFT [88,90,98]. Both Wade et al. [47] and Cruz-Aguilar et al. [95,96] observed a significant improvement in sleep among individuals with mild-to-moderate AD following melatonin supplementation. As a result, the beneficial role of melatonin in AD may be due to its ability to restore disruptions in circadian rhythms. Although Wade et al. [47] did not examine melatonin’s impact on oxidative stress and inflammation biomarkers, several preclinical studies provided evidence that melatonin can reduce inflammation and oxidative stress related to AD. For example, melatonin ameliorated DNA fragmentation and apoptosis in rat hippocampal tissue cultures treated with okadaic acid, a pro-oxidant compound that can generate NFT in rat models [99]. In PC12 cells, agomelatine—an agonist of melatonin receptors MT1 and MT2—attenuated Aβ_25–35_-induced oxidative stress (MDA and ROS) and phosphorylated tau levels in addition to restoring cell viability [100]. Additionally, in rats injected with lipopolysaccharides to induce neuroinflammation, melatonin treatment significantly attenuated inflammation (TNF-α and IL-1β), oxidative stress (MDA and GSH), and acetylcholinesterase activity [101]. As a result, the beneficial impact of melatonin on cognitive function may be due to its ability to reduce oxidative stress and inflammation. Moreover, melatonin may improve cognitive function by reducing the formation of Aβ plaques and NFT. For example, Roy et al. [102] hypothesized that melatonin improved acetylcholine neurotransmission in AD by reducing the formation of Aβ plaques and NFT. The ability of melatonin to prevent Aβ plaques from forming could be related to melatonin contributing to the increased activity of α-secretase and decreased activity of β-secretase [98]. Nevertheless, the results from cell and animal studies do not always translate to humans, and therefore, more research is needed on the role of melatonin in AD and its potential mechanisms.

## 5. Resveratrol

Resveratrol (3,5,4′-trihydroxystillbene) is a polyphenol that can be obtained from various plant sources, such as blackberries, blueberries, bilberries, cranberries, grapes, and peanuts [103,104,105]. A 6-month pilot study indicated that consumption of 72 g of freeze-dried grape powder, compared to a placebo, protected individuals with mild cognitive decline from cerebral metabolic changes and correlated with improved attention and working memory [106]. Additionally, low-to-moderate consumption of red wine, another source of resveratrol, is associated with a lower risk of AD [107,108,109]. Previous preclinical research indicated that resveratrol might possess anti-inflammatory, anti-apoptotic, antioxidant, anticarcinogenic, and cardioprotective properties [110,111]. Furthermore, a previous systematic review [112] indicated that resveratrol might impact cognitive function in AD. As a result, resveratrol is an antioxidant of interest for potentially treating AD. Four articles were identified that examined the impact of resveratrol supplementation on cognitive function in AD.

In the United States, Turner et al. [48] conducted a phase 2 randomized control trial (RCT) among individuals with mild-to-moderate AD. Participants were randomized to receive either resveratrol (*n* = 62) or a placebo (*n* = 55) daily for 52 weeks, starting at a 500 mg dose and increasing it by 500 mg every 13 weeks [48]. While safety and tolerability examination revealed no significant differences between the two groups for neurological examination, physical examination, routine laboratory tests, and vital signs, 95% of the 119 participants experienced an adverse event [48]. There was no significant difference between the groups for the most common adverse events (nausea and diarrhea) [48]. Furthermore, the 36 serious adverse events (27 hospitalizations and three deaths) were determined not to be related to the study or drug [48]. As a result, Turner et al. [48] concluded that resveratrol supplementation was safe and tolerable among individuals with mild-to-moderate AD. After 52 weeks, the placebo group experienced a significantly greater decline in CSF and plasma Aβ_40_ compared to the resveratrol group, but no significant change was observed in CSF and plasma Aβ_42_, CSF tau, CSF phosphorylated tau 181 (p-tau), or plasma glucose and insulin metabolism [48]. A systematic review by Olsson et al. [113] observed no significant differences in the CSF Aβ_40_ for individuals with AD compared to those with MCI. Furthermore, MRIs of participant brain volume revealed a greater decrease in total brain volume among the treatment group [48]. Lastly, Turner et al. [48] observed no significant differences between the groups for ADAS-Cog, Clinical Dementia Rating (CDR)-sum of boxes, MMSE, or NPI but a significantly reduced decline in Alzheimer’s Disease Cooperative Study (ADCS) Activities of Daily Living (ADCS-ADL) among the treatment group. The study had several limitations, and the authors noted that the study might have been underpowered to detect changes in some variables [48].

To understand the mechanisms behind the beneficial role of resveratrol on cognitive function, Moussa et al. [49] examined biomarkers of inflammation at baseline and after 52 weeks in the CSF and plasma of a subset (resveratrol, *n* = 19; placebo, *n* = 19) of Turner et al.’s [48] participants. As reported in the previous study [48], the placebo group experienced a greater decline in ADCS-ADL scores compared to the resveratrol group [49]. Additionally, MMSE declined significantly in the placebo group but not in the resveratrol group [49]. While changes in plasma biomarkers of inflammation were observed, these changes lost significance after adjustment for multiple comparisons [49]. In contrast to Turner et al. [48], Moussa et al. [49] observed a significant decline in CSF Aβ_40_ in the resveratrol group instead of the placebo group after 52 weeks. While the placebo and resveratrol groups experienced a significant decrease in CSF Aβ_42_, the levels were significantly lower in the placebo group [49]. Additionally, matrix metalloprotease (MMP) 9 and CSF macrophage-derived chemokine levels were significantly higher in the resveratrol group compared to the placebo group at 52 weeks [49]. The significant decrease in CSF MMP9 levels indicated that resveratrol might improve cognitive function by reducing the blood-brain barrier’s permeability to inflammation [49].

In a study conducted in the United States, Zhu et al. [50] hypothesized that adding dextrose and malate to a resveratrol supplement would contribute electrons to the metabolism so it could regenerate reduced resveratrol in the brain. Individuals with mild-to-moderate AD taking donepezil were randomized to receive either the intervention (*n* = 17)—resveratrol (5 mg) in combination with dextrose (5 g) and malate (5 g)—or a placebo (*n* = 15) twice daily for a year [50]. Subsequently, one participant withdrew from the intervention group and two from the control group and were excluded from the final analysis [50]. Since none of the adverse events (control group, *n* = 4; intervention group, *n* = 3) were deemed to be related to the study drug, it was concluded that supplementation of 5 mg resveratrol with 5 g dextrose and 5 g malate was safe and tolerable among individuals with AD [50]. No significant differences were observed between the control and intervention groups for ADAS-Cog, ADCS-ADL, ADCS Clinician’s Global Impression of Change (ADCS-CGIC), MMSE, or NPI after one year of treatment [50]. Nevertheless, early discontinuation of recruitment resulted in the study being underpowered to detect a significant change in the primary outcome variable, ADAS-Cog [50]. Therefore, studies with a larger sample size on resveratrol supplementation in combination with dextrose and malate may yield different results.

In China, Fang et al. [51] examined the impact of resveratrol on cognitive function in hospitalized individuals with AD. Participants were randomized to receive either donepezil hydrochloride with (*n* = 45) or without (*n* = 45) 1 g or 2 g of resveratrol for two months [51]. Participants received a 5 mg dose of donepezil for the first month, which could be increased to 10 mg if needed for the last month of the trial [51]. After two months of treatment, the resveratrol group had significantly higher MMSE and lower ADAS-Cog scores, indicating better cognitive function [51]. The resveratrol group also had better measures of living ability, as indicated by the Functional Independence Measure (FIM) [51]. Additionally, the resveratrol group had significantly lower levels of biomarkers of inflammation (IL-6 and TNF-α) [51]. As a result, the study indicated that resveratrol supplementation could potentially improve cognitive function in individuals with AD by reducing inflammation. Nevertheless, the study faced several limitations. For example, the study did not include outcome data comparing individuals in the experimental group who received 1 g versus 2 g of resveratrol. In addition, the generalizability of the study’s results was limited by the small sample size and short duration.

While the mechanisms explaining the beneficial role of resveratrol in AD remain unclear, resveratrol may improve cognitive function by modulating inflammation and oxidative stress. For example, both Moussa et al. [49] and Fang et al. [51] observed changes in markers of inflammation. Moussa et al. [49] observed significantly lower CSF MMP9 levels in individuals treated with AD, indicating that resveratrol might reduce MMP9 levels and, thus, the permeability of the blood-brain barrier to inflammation. Fang et al. [51] observed a significant decline in serum biomarkers of inflammation (IL-6 and TNF-α). Several preclinical studies indicated that the antioxidant and anti-inflammatory properties of resveratrol might involve signaling pathways dependent on AMP-activated protein kinase (AMPK) [114,115] and SIRT1 [116]. For example, Chiang et al. [115] indicated that resveratrol reduced Aβ_1–42_-induced inflammation (IL-1β and TNF-α) and restored cell viability in human neural stem cells by activating AMPK. Kong et al. [117] observed that intraperitoneal resveratrol improved cognitive function and enhanced the gene expression of GPX while decreasing MDA tissue levels in the brains of a mouse model of AD.

## 6. Rosmarinic Acid

First isolated from rosemary (*Rosmarinus officinalis*), rosmarinic acid (labiatenic acid) is an ester of caffeic acid and 3,4-dihydroxyphenyllactic acid [118,119,120]. Other sources of rosmarinic acid include basil, lemon balm, mint, oregano, sage, and thyme [121,122,123]. Recent research indicated that rosmarinic acid possesses antioxidant, antibacterial, antiviral, anti-inflammatory, antihyperglycemic, hepatoprotective, anticancer, cardioprotective, and neuroprotective properties [123,124,125,126]. The antioxidant and anti-inflammatory properties of rosmarinic acid may benefit the central nervous system. For example, two clinical studies showed anxiety- and insomnia-related symptoms improved after supplementation with *Melissa officinalis* (lemon balm) [127] or *Melissa officinalis* and *Nepeta menthoides* [128]. Herrlinger et al. [129] observed an improvement in working memory among individuals with age-associated memory impairment after the daily consumption of a spearmint supplement for 90 days. As a result, rosmarinic acid is an antioxidant of interest in potentially treating and preventing AD, and one clinical trial was identified examining the impact of rosmarinic acid on cognitive function in AD.

In Japan, Noguchi-Shinohara et al. [52] conducted a clinical trial to examine the safety and efficacy of the daily consumption of *Melissa officinalis* extract (containing 500 mg of rosmarinic acid) in patients with mild AD. The first phase of the study included a double-blind trial and randomized participants to receive either *Melissa officinalis* (*n* = 12) or a placebo (*n* = 11) for 24 weeks [52]. The study’s second phase began as an open-label trial, with all participants allocated to the intervention group [52]. While no significant differences in vital signs, physical examination, or neurological examination were observed between the two groups, the placebo group experienced significantly more adverse events than the *Melissa officinalis* group [52]. Therefore, it was concluded that 500 mg of *Melissa officinalis* extract was safe and tolerable in individuals with AD. The study also examined secondary outcomes related to cognitive function and AD-related biomarkers. In the *Melissa officinalis* group, serum rosmarinic acid and its conjugate forms increased from baseline to week 24, but levels in the CSF were undetectable [52]. Noguchi-Shinohara et al. [52] observed a significant improvement in NPI scores among the *Melissa officinalis* group, particularly for the irritability and liability components of the NPI. Nevertheless, there were no significant changes in MMSE, ADAS-Cog, Disability for Dementia (DAD), or CDR scores [52]. Additionally, the researchers observed no significant changes in disease-related biomarkers (CSF Aβ_1–42_, CSF tau, and CSF p-tau) [52]. As a result, the study indicated that *Melissa officinalis* supplementation might improve agitation in individuals with AD but no other markers of cognitive function [52]. Nonetheless, the study’s small sample size and short duration limited the generalizability of the findings. As a result, the study may have been underpowered to detect changes in cognitive function in individuals with AD.

Several in vitro and in vivo studies have examined the potential mechanism of action of rosmarinic acid in AD treatments. Potential mechanisms for improving cognitive function in AD include metal chelation and reducing oxidative stress and inflammation. For example, excess copper can contribute to Aβ plaque formation, and copper-containing Aβ plaques can generate ROS [26]. In a cell culture study, Kola et al. [122] observed that rosmarinic acid protected NIH3T3 cells from copper toxicity by forming a 2:1 complex with cupric copper. Mirza et al. [124] observed a significant improvement in spatial memory in mice injected with the Aβ_1–42_ peptide treated with rosmarinic acid compared to those treated without rosmarinic acid. Furthermore, in a rat model of neuroinflammation, rosmarinic acid treatment attenuated hippocampal neuron loss and damage, short-term working memory decline, inflammation (TNF-α, IL-1β, and IL-6), and markers of oxidative stress (MDA) [121]. As a result, rosmarinic acid may improve cognitive function in AD by reducing oxidative stress and inflammation. Nevertheless, the results from cell and animal studies do not always translate to human beings, and Noguchi-Shinohara et al. [52] observed no changes in measures of cognitive function (MMSE, ADAS-Cog, DAD, or CDR) in individuals treated with rosmarinic acid.

## 7. Carotenoids

Carotenoids represent a large class of lipid-soluble pigment (yellow, orange, and red) molecules such as α- and β-carotene, cryptoxanthin, lutein, lycopene, and zeaxanthin [130,131]. Carotenoids can be classified based on their ability to be converted to vitamin A in the human body as provitamin A (α- and β-carotene and β-cryptoxanthin) and non-provitamin A (lutein, lycopene, and zeaxanthin) [130,131,132]. Carotenoids play a variety of roles in the human body. For example, lutein and zeaxanthin, which can be obtained through the diet from egg yolks, corn, kale, peas, and spinach, are associated with eye health and a reduced risk of age-related macular degeneration [133]. Additionally, carotenoids can function as antioxidants and may help prevent Aβ aggregation and toxicity [134,135]. An observational study observed a reduced risk of AD mortality in adults with either high serum lycopene or lutein and zeaxanthin but no relationship with serum α-carotene, β-carotene, or β-cryptoxanthin [136]. In Japan, a clinical trial indicated that supplementation with polyunsaturated fatty acids (290 mg EPA and 203 mg DHA), lycopene (84 mg), and *Ginkgo biloba* (240 mg) for three years improved cognitive function in community-dwelling older adults compared to controls [137]. As a result, carotenoids are an antioxidant of interest in treating AD. Three articles were identified that examined the impact of carotenoid supplementation on cognitive function in individuals with AD.

In Ireland, Nolan et al. [53] examined the role of carotenoid supplementation on cognitive function in individuals with mild-to-moderate AD and healthy controls. Participants were randomized to one of four groups: AD and carotenoid (*n* = 16); AD and placebo (*n* = 15); healthy control and carotenoid (*n* = 15); or healthy control and placebo (*n* = 16) [53]. The carotenoid supplement contained 10 mg of lutein, 2 mg of zeaxanthin, and 10 mg of meso-zeaxanthin and was consumed daily for six months, while the placebo consisted of sunflower oil [53]. Following six months of treatment, both groups that received the carotenoid supplement experienced a significant improvement in serum lutein, zeaxanthin, and meso-zeaxanthin levels [53]. No significant changes were observed for cognitive function (MMSE) in any of the four groups [53]. As a result, carotenoid supplementation did not improve cognitive function in individuals with AD.

A follow-up study by Nolan et al. [54] supplemented individuals with AD with either the carotenoid supplement (*n* = 12; 10 mg lutein, 2 mg zeaxanthin, and 10 mg meso-zeaxanthin) or the carotenoid supplement in combination with a fish oil supplement (*n* = 13; 430 mg DHA, 90 mg EPA) for 18 months. Compared to the carotenoid-only group, the combined intervention group experienced less functional decline based on medical observations and increased serum lutein and meso-zeaxanthin levels following treatment [54]. Therefore, Nolan et al. [55] conducted a 12-month RCT examining supplementation with carotenoids, fish oil, and vitamin E in individuals with mild-to-moderate AD. Participants were randomized to either the supplement group (*n* = 50; 10 mg lutein, 10 mg meso-zeaxanthin, 2 mg zeaxanthin, 500 mg DHA, 150 mg EPA, and 15 mg α-tocopherol) or a placebo (*n* = 27; sunflower oil) [55]. After participant withdrawal and loss to follow-up, 38 individuals remained in the intervention group and 19 in the placebo group and were included in the final analysis [55]. Following treatment, the intervention group experienced a significant increase in skin carotenoid scores [55]. After controlling for increased dietary carotenoid intake in the intervention group, serum nutrient levels (lutein, meso-zeaxanthin, zeaxanthin, DHA, EPA, and vitamin E) increased significantly compared to the placebo group [55]. Although the intervention group experienced less decline in caregiver-reported patient memory than the placebo group, no significant changes were observed for mean cognitive function as indicated by the MMSE and the Dementia Severity Rating Scale (DSRS) [55]. Upon categorizing participants by dementia severity (DSRS; mild, moderate, and severe), a significant improvement was observed in the intervention group, while the placebo group experienced a significant decline [55]. Lastly, no significant changes were observed for the clinical frailty score or patient- or caregiver-reported perceived quality of life [55]. While the study indicates that carotenoid supplementation combined with fish oil and vitamin E can potentially improve cognitive function in AD, the study was underpowered to detect changes in the MMSE [55]. Therefore, more research is required to examine the impact of carotenoid supplementation on cognitive function in AD.

## 8. Curcumin

Curcumin (1,7-bis-(4-hydroxy-3-methoxyphenyl)-1, 6-heptadiene-3,5-dione), also known as diferuloylmethane, is a component of the spice turmeric, which is derived from the plant *Curcuma longa* [138,139,140,141,142]. Previous research indicates that the consumption of curry, which is rich in curcumin, is associated with greater cognitive performance among older adults [143,144]. Curcumin possesses anti-inflammatory, antioxidant, and metal-chelating properties [140,141,142,145]. Additionally, several preclinical trials indicate that curcumin can attenuate cognitive decline by interacting with Aβ peptides to prevent plaque formation [139]. In fact, curcumin’s ability to interact with Aβ has led to recent interest in using curcumin compounds and derivatives with imaging technology to potentially diagnose AD [146]. An 18-month randomized clinical trial of older dementia-free adults observed an improvement in memory in participants supplemented with curcumin (*n* = 21) compared to a placebo (*n* = 19) [147]. As a result, curcumin is an antioxidant of interest in treating AD. One clinical trial was identified that examined the impact of curcumin supplementation on cognitive function in AD.

In the United States, Ringman et al. [56] examined the role of curcumin supplementation in individuals with mild-to-moderate AD. Participants were randomized to receive either 2 g of curcumin (*n* = 12), 4 g of curcumin (*n* = 12), or a placebo daily for 24 weeks [56]. After a few weeks, the RCT was converted to an open-label trial, and the placebo group was randomized to either 2 g or 4 g of curcumin for 24 more weeks. Following participant withdrawal, nine individuals remained in the 2 g curcumin group, 10 in the 4 g curcumin group, and 11 in the placebo group [56]. While Ringman et al. [56] observed no significant differences between the three groups for measures of cognitive function (MMSE, ADAS-Cog, ADCS-ADL, NPI) after 24 weeks of treatment, the authors noted a non-significant trend towards worsening cognitive function (MMSE, ADAS-Cog) in the curcumin groups combined compared to the placebo group. Additionally, no significant differences were observed between the three groups for biomarkers of AD (plasma Aβ_40_ and Aβ_42_; CSF Aβ_42_, t-tau, and p-tau) and CSF isoprostanes [56]. As a result, the study indicated that curcumin supplementation did not improve cognitive function in individuals with AD. Nevertheless, the study had several limitations, including a small sample size. Additionally, research [145,148,149] indicates that curcumin has low bioavailability, and Ringman et al. [56] were unable to detect curcuminoids in the CSF, though a slight increase was observed in the plasma of treated individuals. While Small et al. [147] observed a beneficial effect of curcumin supplementation on memory in older adults, the study’s participants were dementia-free, and the study drug had been formulated to increase curcumin absorption in the gastrointestinal tract. Therefore, future research using different curcumin formulations and larger sample sizes may yield different results.

## 9. Vitamin E

Vitamin E is a fat-soluble vitamin consisting of eight different isoforms, including α-, β-, γ-, and δ-tocopherols and tocotrienols [150]. Dietary sources of vitamin E may vary in the amount of a particular isoform, with some plant-based sources being richer in one isoform over another [150,151]. For example, α-tocopherol—the most common isoform of vitamin E in the human body—is more abundant than other isoforms of vitamin E in hazelnuts, almond oil, and sunflower oil [150,151,152]. Vitamin E is an antioxidant that can function as a free radical scavenger to prevent the peroxidation of polyunsaturated fatty acids in the lipid bilayer of cells [153]. A meta-analysis by Lopes da Silva et al. [154] reported significantly lower plasma vitamin E levels in individuals with AD compared to controls. In 1997, a study published by Sano et al. [155] indicated that supplementation with 2000 IU of vitamin E reduced the progression to either death, institutionalization, loss of ADL, or severe dementia in individuals with moderately severe AD compared to a placebo. As a result, vitamin E is an antioxidant of interest in potentially treating AD. Four clinical trials were identified that examined the impact of vitamin E supplementation on cognitive function in individuals with AD.

In Germany, Arlt et al. [57] conducted an open-label trial to examine the impact of combined vitamin E and C supplementations in individuals with mild-to-moderate AD who take cholinesterase inhibitors. While the control group (*n* = 11) received standard care, the intervention group (*n* = 12) received vitamin E (400 IU) and vitamin C (1000 mg) daily for a year [57]. At baseline, the intervention group was composed of younger participants (mean age 67.7 ± 7.2 vs. 73.7 ± 5.3) with an earlier age at diagnosis (mean age 63.9 ± 7.6 vs. 71.1 ± 5.5) than the control group [57]. After one month of treatment, the intervention group experienced a significant increase in CSF α-tocopherol of 42% and a 14% increase in ascorbate, which remained relatively stable for the duration of the trial [57]. Additionally, Arlt et al. [57] observed a significant reduction in the rate of CSF oxidation after one year but not after one month. Despite these changes, no significant differences emerged between the two groups for cognitive function (MMSE, immediate and delayed word recall, word fluency, and the trail-making test) at baseline or after one year of treatment [57]. While the difference between the two groups for mean change in MMSE scores after one year was not statistically significant (intervention group, −2.08 ± 3.73; control group, −1.64 ± 2.26), the decline in MMSE scores experienced by the intervention group was statistically significant. Nevertheless, the study’s data is limited by its small sample size, open-label design, and absence of data on oxidative stress in the control group. Overall, the study does not support the beneficial role of vitamin E supplementation in combination with vitamin C in improving cognitive function in AD.

In the United States, Galasko et al. [58] examined the impact of an antioxidant supplement containing vitamin E (α-tocopherol) in comparison to supplementation with coenzyme Q alone or a placebo among individuals with mild-to-moderate AD. Participants were randomized to receive either an antioxidant supplement (800 IU vitamin E, 500 mg vitamin C, and 900 mg α-lipoic acid; *n* = 26), 400 mg coenzyme Q three times a day (*n* = 26), or a placebo (*n* = 26) for 16 weeks [58]. After excluding participants who withdrew from the study, 24 individuals remained in the antioxidant supplement group, 20 in the coenzyme Q group, and 18 in the placebo group and were included in the final analysis [58]. Compared to the coenzyme Q and placebo groups, the combined antioxidant group experienced a significant decline in cognitive function (MMSE) following treatment [58]. As a result, vitamin E supplementation combined with vitamin C and α-lipoic acid may negatively impact cognition [58]. Additionally, F2-isoprostane levels significantly decreased after treatment in the combined antioxidant group, indicating decreased oxidative stress in the central nervous system [58]. No significant differences emerged between the three groups for ADAS-ADL or CSF biomarkers (Aβ_42_, t-tau, or p-tau) [58]. Therefore, the study observed either no impact (coenzyme Q) or a negative impact (combined antioxidant group) of antioxidant supplementation on cognitive function in individuals with AD.

In the United States, Dysken et al. [59] examined the impact of vitamin E supplementation (α-tocopherol) with or without memantine on cognitive function in individuals with mild-to-moderate AD. Participants were randomized to one of four groups: 2000 IU vitamin E (*n* = 152), 20 mg memantine (*n* = 155), vitamin E and memantine (*n* = 154), or placebo (*n* = 152) daily [59]. Participants were scheduled for a follow-up every six months for four years, with the average participant being followed for 2.27 years [59]. After excluding participants without follow-up data, the study included 140 participants from the vitamin E group, 142 from the memantine group, 139 from the combination group, and 140 from the placebo group in the final analysis. Compared to the placebo group, participants receiving vitamin E alone experienced significantly less decline in ADCS-ADL scores. The average decline in ADCS-ADL was reduced by 3.15 units, indicating a clinically significant attenuation in the loss of functional ability. Although the placebo group experienced a greater decline in ADCS-ADL scores than the combined treatment group, the result was not statistically significant. Additionally, caregivers spent significantly less time (mean = 2.17 h) assisting participants (Caregiver Activity Survey) in the vitamin E group compared to the memantine group [59]. No other significant differences were detected for the MMSE, ADAS-Cog, NPI, or Dependence Scale. As a result, the trial indicated that 2000 IU of vitamin E without memantine might prevent an individual with AD’s ADL from declining and alleviate the time caregivers spend assisting individuals. In terms of safety, Dysken et al. [59] observed no significant differences between the vitamin E and placebo groups for adverse events, serious adverse events, or mortality. Conversely, significantly more serious adverse events were reported in the memantine and combination groups than in the placebo group [59]. While the study indicated that 2000 IU of vitamin E is safe for individuals with AD, the study’s generalizability is limited by the predominantly Caucasian (86%) male (97%) sample [59]. Additionally, larger sample sizes may be required to detect changes in other markers of cognitive function (MMSE and ADAS-Cog).

Furthermore, in the United States, Remington et al. [60] examined the impact of a nutraceutical supplement containing vitamin E in individuals with AD. Participants were randomized to receive either a supplement containing 400 μg folic acid, 30 IU α-tocopherol, 6 μg vitamin B_12_, 400 mg S-adenosyl methionine, 600 mg N-acetyl cysteine, and 500 mg acetyl-L-carnitine (*n* = 86) or a placebo (*n* = 57) twice daily for three to six months, followed by all participants being allocated to the intervention group for another six months [60]. After excluding participants lost to follow-up, 84 individuals remained in the intervention group and 57 in the placebo group [60]. Following three months of supplementation, participants in the intervention group had a significant improvement in cognitive function (Dementia Rating Scale) but no significant changes in NPI or ADCS-ADL scores [60]. Following the end of the open-label portion of the trial, both participants initially allocated to the intervention or placebo groups experienced either an improvement or maintenance of cognitive function [60]. Remington et al. [156] also reported that a cohort of 24 individuals with AD who consumed the neutraceutical supplement under open-label conditions for one year experienced no significant changes in the Dementia Rating Scale, NPI, or ADCS-ADL. The authors indicated that this lack of change suggested that the neutraceutical supplement prevented cognitive decline in participants [156]. As a result, the authors concluded that the nutraceutical supplement could improve cognitive function or prevent cognitive decline in individuals with AD [60,156]. Nevertheless, the study had several limitations, including small sample sizes and a lack of diversity among participants. Additionally, the inclusion of other nutrients besides vitamin E prevented researchers from drawing conclusions about the benefits of vitamin E supplements in individuals with AD.

While the mechanisms explaining the benefits of vitamin E in AD are unclear, vitamin E may improve cognitive function in individuals with AD by reducing inflammation and oxidative stress [153,157]. In a study by Giraldo et al. [158], vitamin E treatment attenuated p38 activation and tau phosphorylation in Aβ_1–42_-treated cortical neurons. Vitamin E supplementation also reduced p38 activation but not tau phosphorylation in the hippocampus of APP/PS1 mice [158]. Additionally, Ibrahim et al. [159] observed that vitamin E supplementation attenuated cognitive decline and Aβ accumulation in the cortex but not the hippocampus of APP/PS1 mice. Desrumaux et al. [160] examined the impact of vitamin E supplementation in phospholipid transfer protein (PLTP) knock-out mice with intracerebrovascular injection of Aβ_25–35_. PLTP is involved in the transfer of α-tocopherol throughout the body, and its deficiency results in increased circulating vitamin E and decreased vitamin E concentrations in the brain [160]. Vitamin E supplementation significantly restored vitamin E levels in the brains of PLTP knock-out mice and reduced oxidative stress (the cerebral oxidative stress index) and Aβ_1–42_ accumulation but did not affect inflammation (IL-6 and TNF-α) [160]. Nevertheless, the results from cell and animal studies do not always translate to human beings, and Dysken et al. [59] and Sano et al. [155] observed no changes in measures of cognitive function (MMSE) in individuals supplemented with vitamin E.

## 10. Coenzyme Q

Coenzyme Q_10_ (2,3-dimethoxy-5-methyl-6-multiprenyl-1,4-benzoquinone) is known as ubiquinone in its oxidized form and as ubiquinol in its reduced form [161,162,163]. Coenzyme Q_10_ can be synthesized by the human body, where it is involved in the electron transport chain of the mitochondria [163,164]. Additionally, it can be obtained from dietary sources, including fatty fish, spinach, and nuts, or from supplements [163]. Coenzyme Q_10_ is an antioxidant of interest in treating AD due to its free-radical scavenging capability and role in the regeneration of vitamins C and E [165]. Two systematic reviews and meta-analyses examining coenzyme Q_10_ supplementation and markers of oxidative stress observed that coenzyme Q_10_ significantly reduces MDA, increases superoxide dismutase and TAC, and does not impact catalase, GPX, or GSH [161,162]. While preclinical studies indicate that coenzyme Q_10_ is potentially therapeutic in dementia treatment, clinical studies on coenzyme Q_10_ supplementation published before 2012 have yielded mixed results [165]. Furthermore, a systematic review of three studies observed no significant difference between the coenzyme Q_10_ serum/plasma levels of individuals with AD and controls [165]. Nevertheless, coenzyme Q_10_ remains an antioxidant of interest in treating AD. One clinical trial was identified that examined the impact of coenzyme Q_10_ supplementation on cognitive function in AD.

As mentioned earlier, Galasko et al. [58] examined the impact of coenzyme Q (400 mg, three times a day) in comparison to an antioxidant supplement (800 IU vitamin E, 500 mg vitamin C, and 900 mg α-lipoic acid) or a placebo for 16 weeks in individuals with mild-to-moderate AD. While the combined supplementation increased the rate of cognitive decline (MMSE), coenzyme Q had no significant impact [58]. Coenzyme Q did not impact ADAS-ADL scores or CSF biomarkers (F2-isoprostane, Aβ_42_, t-tau, and p-tau) [58]. While the study is already limited by its small sample size, its data could also be impacted by the type of coenzyme Q supplement used. For example, Mantle et al. [164] noted that the bioavailability of coenzyme Q_10_ could vary based on the formulation of the supplement. In light of the promising role of coenzyme Q_10_ being beneficial for potentially treating neurodegenerative diseases and clinical studies on its role in AD yielding mixed results, more research is needed to determine the impact of coenzyme Q_10_ on cognitive function and oxidative stress in AD [163,165].

## 11. Summary and Conclusions

**Figure 1:**
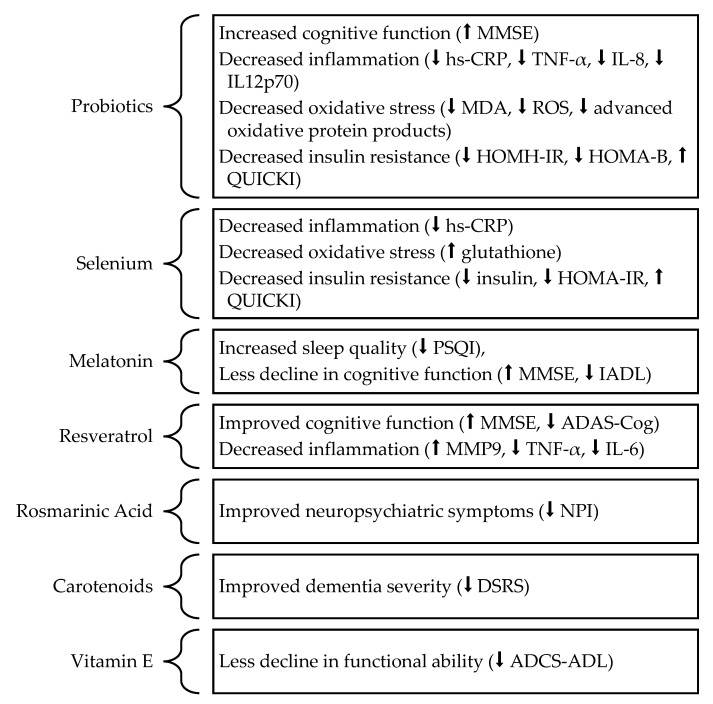
The potential beneficial effects of different antioxidant supplements in individuals with AD. Upward arrows (⬆) indicate that the beneficial relationship results from the indicated value increasing, while downward arrows (⬇) indicate that the beneficial relationship results from the indicated value decreasing. Abbreviations: AD, Alzheimer’s disease; ADAS-Cog, Alzheimer’s Disease Assessment Scale-cognitive; ADCS, Alzheimer’s Disease Cooperative Study; ADCS-ADL, Alzheimer’s Disease Cooperative Study Activities of Daily Living; DSRS, Dementia Severity Rating Scale; HOMA-B, Homeostatic Model of Assessment for B-cell Function; HOMA-IR, Homeostatic Model of Assessment for Insulin Resistance; hs-CRP, high-sensitivity C-reactive protein; IADL, Instrumental Activities of Daily Living; IL, Interleukin; MDA, Malondialdehyde; MMP, Matrix Metalloprotease; MMSE, Mini-Mental State Examination; NPI, Neuropsychiatric Inventory; PSQI, Pittsburgh Sleep Quality Index; QUICKI, Quantitative Insulin Sensitivity Check Index; ROS, Reactive Oxygen Species; TNF-α, Tumor Necrosis Factor α.

In summary, this review article examined the results of clinical trials involving supplementation with antioxidants on cognitive function in individuals with AD (Figure 1). First, though Agahi et al. [41] and Leblhuber et al. [42] observed no relationship between probiotic supplementation and cognitive function, the studies by Akbari et al. [40] and Ton et al. [43] observed that probiotic supplementation could improve cognitive function in individuals with AD. The authors also provided evidence that probiotics may improve cognitive function by reducing oxidative stress and inflammation [40,43]. Second, while Maplas et al. [44] observed no benefits of selenium supplementation on cognitive function in individuals with AD, Cardoso et al. [45] observed that selenium supplementation prevented cognitive decline after grouping the study’s participants into selenium ‘responsive’ and ‘non-responsive’ groups based on changes in CSF and plasma selenium concentrations. Tamtaji et al. [46] also observed a significant improvement in cognitive function in individuals with AD supplemented with both selenium and probiotics but not selenium alone. Since the trial did not include a probiotic-only group, it is unclear whether the beneficial effects resulted from probiotic supplementation or the combination of the two supplements. Third, Wade et al. [47] observed that melatonin supplementation prevented cognitive decline in individuals with AD. Fourth, in contrast to Turner et al. [48] and Zhu et al. [50], Fang et al. [51] observed a beneficial impact of resveratrol supplementation on cognitive function in individuals with AD. Additionally, both Turner et al. [48] and Fang et al. [51] observed a beneficial impact of resveratrol supplementation on functional ability. Fifth, though Noguchi-Shinohara et al. [52] observed no significant changes in measures of cognitive function following supplementation with rosmarinic acid, the study’s participants experienced a significant improvement in measures of irritability and liability. Sixth, the studies by Nolan et al. [53,54,55] observed little benefit of carotenoid supplementation on cognitive function in individuals with AD, though Nolan et al. [55] observed beneficial changes in the AD severity category and caregiver-reported patient memory. Seventh, Ringman et al. [56] observed no relationship between curcumin supplementation and cognitive function, though the authors’ noted a non-significant trend toward worsening cognitive function. Eight, while Dysken et al. [59] observed no significant benefits of vitamin E on cognitive function, participants receiving vitamin E supplementation experienced significantly less decline in ADL than controls. In contrast, Remington et al. [60] observed a significant improvement in cognitive function but no difference in ADL in individuals with AD receiving a vitamin E supplement compared to controls. Nevertheless, the vitamin E supplement in the study by Remington et al. [60] included a lower dose of vitamin E and contained other nutraceutical compounds (folic acid, vitamin B_12_, S-adenosyl methionine, N-acetyl cysteine, and acetyl-L-carnitine). Arlt et al. [57] observed no relationship between vitamin E and C supplementation and cognitive function. Galasko et al. [58] also observed a significant decline in cognitive function in individuals receiving a vitamin E-containing supplement compared to either coenzyme Q or a placebo. As a result, the comparability of the four studies is limited. Overall, the ability of antioxidant supplements to improve cognitive function in individuals with AD remains unclear.

As of January 2023, there are three ongoing clinical trials examining probiotic supplementation (NCT05521477, NCT04606420, and NCT05145881) in individuals with MCI or AD registered with clinicaltrials.gov [166,167,168]. Additionally, two ongoing clinical trials examining melatonin supplementation (NCT03954899 and NCT05543681) and another examining vitamin E supplementation (NCT05343611) and one examining coenzyme Q, probiotics, and curcumin supplementation (NCT04606420) in individuals with MCI or AD are registered [167,169,170,171]. There are no active clinical trials on selenium, resveratrol, rosmarinic acid, or carotenoid supplementation in AD.

In conclusion, this review article examined the results of clinical trials involving supplementation with antioxidants on cognitive function in individuals with AD. While the studies included in this review did not provide much evidence for the beneficial role of antioxidant supplements on cognitive function in individuals with AD, the results varied from antioxidant to antioxidant among trials examining the same antioxidant. Furthermore, the studies face several limitations, including short trial durations, small sample sizes, and a lack of diversity among study participants. As a result, more research is required to examine the impact of antioxidant supplementation on cognitive function in individuals with AD.

## Figures and Tables

**Table 1:** Summary of Clinical Trials on Antioxidant Supplementation in Individuals with AD.

Authors	Year Published	Location	Participants	Intervention	Duration of Treatment Period	Main Results	Trial Numbers
Akarbi et al. [40]	2016	Iran	AD	200 mL/day of milk either with (*n* = 30) or without (*n* = 30) probiotic supplementation (2 × 10^9^ CFU/g of *Lactobacillus acidophilus*, *Lactobacillus casei*, *Lactobacillus fermentum*, and *Bifidobacterium bifidum*).	12 weeks	Compared to the control group, the probiotic group experienced a significant improvement in MMSE scores, a decline in hs-CRP, MDA, HOMA-IR, HOMA-B, and increased QUICKI.	IRCT201511305623N60 ^c^
Agahi et al. [41]	2018	Iran	AD	Placebo (*n* = 30) or a probiotic (*n* = 30) consisting of two capsules (3 × 10^9^ each of either *Lactobacillus fermentum*, *Lactobacillus plantarum*, and *Bifidobacterium lactis* or *Lactobacillus acidophilus*, *Bifidobacterium bifidum*, and *Bifidobacterium longum*) every other day.	12 weeks	Either no or negligible significant differences were reported for indicators of cognitive function (TYM), oxidative stress (TAC, MDA, NO, total glutathione, and 8-hydroxy-2′-deoxyguanosine), and inflammation (TNF-α, IL-6, and IL-10).	IRCT2017061534549N1 ^c^
Leblhuber et al. [42]	2018	Austria	AD	3 g of a probiotic supplement containing *Lactobacillus casei*, *Lactococcus lactis*, *Lactobacillus acidophilus*, *Bifidobacterium lactis*, *Lactobacillus paracasei*, *Lactobacillus plantarum*, *Bifidobacterium lactis*, *Bifidobacterium bifidum*, and *Lactobacillus salivarius* (*n* = 20)	4 weeks	No significant changes in cognitive function (MMSE, clock drawing test). Fecal *Faecalibacterium prausnitzii* RNA increased, with no change in *Clostridium* cluster I or *Akkermansia muciniphila* RNA. Fecal zonulin significantly declined, and serum kynurenine increased.	
Ton et al. [43]	2020	Brazil	Probable AD	2 mL/kg/day kefir-fermented milk containing *Acetobacter aceti*, *Acetobacter* sp., *Candida famata*, *Candida krusei, Enterococcus faecium*, *Lactobacillus delbrueckii delbrueckii*, *Lactobacillus fermentum*, *Lactobacillus fructivorans*, *Lactobacillus kefiranofaciens*, and *Leuconostoc* spp. (*n* = 16).	90 days	Participants experienced a significant improvement in MMSE scores, inflammation (TNF-α, IL-8, and IL12p70), and oxidative stress (ROS, advanced oxidative protein products, and NO).	
Malpas et al. [44]	2016	Australia	Mild-to-moderate AD	Supranutrional (10 mg sodium selenate; *n* = 20), nutritional (0.32 mg sodium selenate; *n* = 10), or a placebo (*n* = 10) three times a day.	24 weeks	Supplementation with supranutritional selenate was tolerable and safe. No significant changes in AD-related CSF biomarkers (phosphorylated tau, t-tau, and Aβ_1–42_) or cognitive function (MMSE, ADAS-Cog, COWAT, CFT, OCL, IDN, and DET).	ACTRN12611001200976 ^d^
Cardoso et al. [45] ^a^	2019	Australia	Mild-to-moderate AD	Supranutrional (10 mg sodium selenate; *n* = 20), nutritional (0.32 mg sodium selenate; *n* = 10), or a placebo (*n* = 10) three times a day.	24 weeks	There was no difference in the number of drug-related adverse events between the responsive and non-responsive groups. Compared to non-responsive individuals, participants in the responsive group did not experience a significant decline in MMSE. No other measures of cognitive function (ADAS-Cog, COWAT, CFT, OCL, IDN, and DET) were significant.	ACTRN12611001200976 ^d^
Tamtaji et al. [46]	2019	Iran	Institutionalized individuals with AD	200 μg selenium with (*n* = 30) and without (*n* = 30) a probiotic (2 × 10^9^ CFU of *Lactobacillus acidophilus*, *Bifidobacterium bifidum*, and *Bifidobacterium longum*) supplement or a placebo (*n* = 30) daily.	12 weeks	The selenium-only group experienced a significant improvement in markers of inflammation (hs-CRP), oxidative stress (glutathione), and insulin resistance (insulin, HOMA-IR, and QUICKI) compared to the placebo group. Compared to the placebo and selenium-only groups, the combination group experienced a significant improvement in cognitive function (MMSE), inflammation (hs-CRP), oxidative stress (TAC and glutathione), and insulin resistance (insulin, HOMA-IR, and QUICKI).	IRCT20170612034497N5 ^c^
Wade et al. [47]	2014	United Kingdom, United States	Mild-to-moderate AD	2 mg prolonged-release melatonin supplement (*n* = 39) or a placebo (*n* = 34) daily.	24 weeks	Supplementation with prolonged-release melatonin was tolerable and safe. The melatonin group experienced an improvement in overall sleep quality (PSQI), less decline in cognitive function (MMSE), and an improvement in IADL. No changes were observed for ADAS-Cog.	
Turner et al. [48]	2015	United States	Mild-to-moderate AD	Resveratrol (*n* = 62) or placebo (*n* = 55) daily, starting at a 500 mg dose and increasing by 500 mg every 13 weeks.	52 weeks	Supplementation with resveratrol was safe and tolerable. Compared to the placebo group, the resveratrol group experienced less decline in CSF, plasma Aβ_40_, and ADCS-ADL after 52 weeks. No changes were observed for AD-related biomarkers (CSF Aβ_42_, plasma Aβ_42_, CSF tau, and CSF p-tau), cognitive function (ADAS-Cog, CDR-sum of boxes, MMSE, and NPI), or plasma glucose and insulin metabolism.	NCT01504854 ^e^
Moussa et al. [49] ^b^	2017	United States	Mild-to-moderate AD	Resveratrol (*n* = 62) or placebo (*n* = 55) daily, starting at a 500 mg dose and increasing by 500 mg every 13 weeks.	52 weeks	ADCS-ADL and MMSE scores significantly declined in the placebo group. CSF Aβ_40_ declined significantly in the resveratrol group. Compared to the placebo group, the resveratrol group experienced significantly less decline in CSF Aβ_42_ and greater increase in MMP9.	NCT01504854 ^e^
Zhu et al. [50]	2018	United States	Mild-to-moderate AD	A supplement containing 5 mg resveratrol, 5 g dextrose, and 5 g malate (*n* = 17) or a placebo (*n* = 15).	1 year	No significant differences between groups for ADAS-Cog, ADCS-ADL, ADCS-CGIC, MMSE, or NPI were observed.	NCT00678431 ^e^
Fang et al. [51]	2022	China	Hospitalized individuals with AD	Donepezil hydrochloride with (*n* = 45) or without (*n* = 45) 1 g or 2 g of resveratrol daily.	2 months	The resveratrol group had significantly improved cognitive function (MMSE and ADAS-Cog), living ability (FIM), and biomarkers of inflammation (IL-6 and TNF-α).	
Noguchi-Shinohara et al. [52]	2020	Japan	Mild AD	*Melissa officinalis* extract containing 500 mg of rosmarinic acid (*n* = 12) or a placebo (*n* = 11).	24 weeks	Supplementation with *Melissa officinalis* was safe and tolerable. Compared to the placebo group, the *Melissa officinalis* group experienced a significant improvement in NPI. No significant changes were observed for cognitive function (MMSE, ADAS-Cog, and CDR), functional ability (DAD), or CSF AD-related biomarkers (Aβ_1–42_, tau, and p-tau).	UMIN000007734 ^f^
Nolan et al. [53]	2015	Ireland	Mild-to-moderate AD Healthy controls	Four groups: AD and carotenoid (*n* = 16; 10 mg lutein, 2 mg zeaxanthin, 10 mg meso-zeaxanthin); AD and placebo (*n* = 15); healthy control and carotenoid (*n* = 15); healthy control and placebo (*n* = 16).	6 months	Both groups receiving the carotenoid supplement experienced an increase in serum lutein, zeaxanthin, and meso-zeaxanthin levels. No significant changes were observed for cognitive function (MMSE).	
Nolan et al. [54]	2018	Ireland	AD	Carotenoid supplement (*n* = 12; 10 mg lutein, 2 mg zeaxanthin, 10 mg meso-zeaxanthin) or the carotenoid supplement in combination with a fish oil supplement (*n* = 13; 430 mg DHA, 90 mg EPA)	18 months	Compared to the carotenoid-only group, the combined intervention group experienced less functional decline based on medical observations and a greater increase in serum lutein and meso-zeaxanthin levels.	
Nolan et al. [55]	2022	Ireland	Mild-to-moderate AD	Supplement with 10 mg lutein, 10 mg meso-zeaxanthin, 2 mg zeaxanthin, 500 mg DHA, 150 mg EPA, and 15 mg α-tocopherol (*n* = 50) or a placebo (*n* = 27)	12 months	Compared to the placebo group, the intervention group experienced a significant increase in serum nutrient levels (lutein, meso-zeaxanthin, zeaxanthin, DHA, EPA, and vitamin E). No significant changes were observed for cognitive function (MMSE and DSRS). Categorizing participants by dementia severity (DSRS), a significant improvement was observed in the intervention group, while the placebo group experienced a significant decline.	ISRCTN11892249 ^g^
Ringman et al. [56]	2012	United States	Mild-to-moderate AD	RCT: 2 g curcumin (*n* = 12), 4 g curcumin (*n* = 12), or a placebo (*n* = 12) daily. Open-label: the placebo group was randomized to either 2 g or 4 g of curcumin.	24 week RCT followed by 24 week open-label period.	No significant between-group differences for measures of cognitive function (MMSE, ADAS-Cog, ADCS-ADL, and NPI), AD biomarkers (plasma Aβ_40_ and Aβ_42_; CSF Aβ_42_, t-tau, and p-tau), or isoprostanes.	NCT00099710 ^e^
Arlt et al. [57]	2012	Germany	Mild-to-moderate AD	Standard care (*n* = 11) or supplementation (*n* = 12) with 400 IU vitamin E and 1000 mg vitamin C daily.	1 year	No significant difference between groups for cognitive function (MMSE, immediate and delayed word recall, word fluency, trail-making test). The intervention group experienced a significant increase in CSF α-tocopherol and ascorbate and a significant decrease in the rate of CSF oxidation.	
Galasko et al. [58]	2012	United States	Mild-to-moderate AD	A supplement containing 800 IU vitamin E, 500 mg vitamin C, and 900 mg α-lipoic acid (*n* = 26); 400 mg coenzyme Q (*n* = 26) three times a day; or a placebo (*n* = 26).	16 weeks	Compared to the other groups, the combined antioxidant group experienced a significant decline in cognitive function (MMSE) and oxidative stress (F2-isoprostane). No significant between-group differences emerged for ADAS-ADL or CSF biomarkers (Aβ_42_, t-tau, or p-tau).	NCT00117403 ^e^
Dysken et al. [59]	2014	United States	Mild-to-moderate AD	2000 IU vitamin E (*n* = 152), 20 mg memantine (*n* = 155), vitamin E and memantine (*n* = 154), or placebo (*n* = 152) daily.	6 months to 4 years	Supplementation with vitamin E was safe and tolerable. Compared to the placebo group, participants receiving vitamin E alone experienced significantly less decline in ADCS-ADL scores. No significant differences were detected for MMSE, ADAS-Cog, NPI, or Dependence Scale.	NCT00235716 ^e^
Remington et al. [60]	2015	United States	Individuals with AD	A supplement containing 400 μg folic acid, 30 IU α-tocopherol, 6 μg vitamin B_12_, 400 mg S-adenosyl methionine, 600 mg N-acetyl cysteine, and 500 mg acetyl-L-carnitine (*n* = 86) or placebo (*n* = 57) twice daily.	3 to 6 months	After three months, the intervention group had a significant improvement in cognitive function (Dementia Rating Scale), but no significant changes in NPI or ADCS-ADL scores.	NCT01320527 ^e^

Abbreviations: Aβ, Amyloid Beta; AD, Alzheimer’s disease; ADAS-Cog, Alzheimer’s Disease Assessment Scale-cognitive; ADCS, Alzheimer’s Disease Cooperative Study; ADCS-ADL, Alzheimer’s Disease Cooperative Study Activities of Daily Living; ADCS-CGIC, Alzheimer’s Disease Cooperative Study Clinician’s Global Impression of Change; CDR, Clinical Dementia Rating; CFT, Category Fluency Test; CSF, Cerebral Spinal Fluid; COWAT, Controlled Oral Word Association Test; DAD, Disability Assessment for Dementia; DET, Detection Reaction Time Task; DHA, Docosahexaenoic Acid; DSRS, Dementia Severity Rating Scale; EPA, Eicosapentaenoic Acid; FIM, Functional Independence Measure; HOMA-B, Homeostatic Model of Assessment for B-cell Function; HOMA-IR, Homeostatic Model of Assessment for Insulin Resistance; hs-CRP, high-sensitivity C-reactive protein; IADL, Instrumental Activities of Daily Living; IDN, Identification Reaction Time Task; IL, Interleukin; MDA, Malondialdehyde; MMP, Matrix Metalloprotease; MMSE, Mini-Mental State Examination; n, sample size; NO, Nitric Oxide; NPI, Neuropsychiatric Inventory; OCL, One-Card Learning Memory Task; PSQI, Pittsburgh Sleep Quality Index; p-tau, Phosphorylated Tau 181; QUICKI, Quantitative Insulin Sensitivity Check Index; RCT, Randomized Controlled Trial; RNA, Ribonucleic Acid; ROS, Reactive Oxygen Species; TAC, Total Antioxidant Capacity; TNF-α, Tumor Necrosis Factor α; t-tau, Total Tau; TYM, Test Your Memory. ^a.^ Participants from the study by Maplas et al. [44] were regrouped as ‘responsive’ and ‘non-responsive’ to sodium selenate based on an increase in serum (responsive, *n* = 17; non-responsive, *n* = 18) and CSF (responsive, *n* = 12; non-responsive, *n* = 14) selenium three times that of baseline levels [45]. ^b.^ Moussa et al. [49] examined biomarkers of inflammation in a subset (resveratrol, *n* = 19; placebo, *n* = 19) of the Turner et al. [48] study’s participants. ^c.^ Iranian Registry of Clinical Trials (irct.ir). ^d.^ Australian New Zealand Clinical Trials Registry (anzctr.org.au). ^e.^ United States (clinicaltrials.gov). ^f.^ University Hospital Medical Information Network Clinical Trials Registry (umin.ac.jp/ctr). ^g.^ International Traditional Medicine Clinical Trial Registry (isrctn.com).

## Abbreviations

Aβ, Amyloid Beta; AD, Alzheimer’s disease; ADAS-Cog, Alzheimer’s Disease Assessment Scale-cognitive; ADCS, Alzheimer’s Disease Cooperative Study; ADCS-ADL, Alzheimer’s Disease Cooperative Study Activities of Daily Living; ADCS-CGIC, Alzheimer’s Disease Cooperative Study Clinician’s Global Impression of Change; AMPK, AMP-Activated Protein Kinase; APP, Amyloid Precursor Protein; CDR, Clinical Dementia Rating; CFT, Category Fluency Test; CSF, Cerebral Spinal Fluid; COWAT, Controlled Oral Word Association Test; DAD, Disability Assessment for Dementia; DET, Detection Reaction Time Task; DHA, Docosahexaenoic Acid; DNA, Deoxyribonucleic Acid; DSRS, Dementia Severity Rating Scale; EPA, Eicosapentaenoic Acid; FIM, Functional Independence Measure; GPX, Glutathione Peroxidase; GSH, glutathione; HOMA-B, Homeostatic Model of Assessment for B-cell Function; HOMA-IR, Homeostatic Model of Assessment for Insulin Resistance; hs-CRP, high-sensitivity C-reactive protein; IADL, Instrumental Activities of Daily Living; IDN, Identification Reaction Time Task; IL, Interleukin; MCI, Mild Cognitive Impairment; MDA, Malondialdehyde; MMP, Matrix Metalloprotease; MMSE, Mini-Mental State Examination; MRI, Magnetic Resonance Imaging; n, sample size; NFT, Neurofibrillary Tau Tangles; NO, Nitric Oxide; NPI, Neuropsychiatric Inventory; OCL, One-Card Learning Memory Task; PLTP, Phospholipid Transfer Protein; PSQI, Pittsburgh Sleep Quality Index; PS1, Presenilin 1; p-tau, Phosphorylated Tau 181; QUICKI, Quantitative Insulin Sensitivity Check Index; RNA, Ribonucleic Acid; ROS, Reactive Oxygen Species; SIRT1, Sirtuin1; TAC, Total Antioxidant Capacity; TNF-α, Tumor Necrosis Factor α; t-tau, Total Tau; TYM, Test Your Memory.
